# Analysis of the Effectiveness of Second Attempt Endoscopic Retrograde Cholangiopancreatography (ERCP) 24 Hours (Second Day) After Primary Failure

**DOI:** 10.7759/cureus.53405

**Published:** 2024-02-01

**Authors:** Dilaram Khan, Inayat Ullah, Mohammad Kashif

**Affiliations:** 1 Gastroenterology, Lady Reading Hospital, Peshawar, PAK; 2 General Medicine, Lady Reading Hospital, Peshawar, PAK

**Keywords:** fluoroscope, effectiveness, second ercp, failed biliary cannulation, needle knife, dual wire technique, second attempt, duedenoscope

## Abstract

Background: Endoscopic retrograde cholangiopancreatography (ERCP) is a minimally invasive intervention that has established itself as the gold standard therapeutic option for various pancreaticobiliary conditions. Deep cannulation of the common bile duct (CBD) is essential in ERCP. However, cannulation is not possible in approximately 20% of the cases with the usual techniques even when performed by highly trained professionals or at major healthcare institutions. In case of failure on the first attempt, alternative choices include redoing the procedure (on the second attempt) or moving on to more proficient endoscopic methods such as endoscopic ultrasound (EUS) or radiology-aided techniques (rendezvous procedures), totally percutaneous approaches, or surgical treatments.

Objective: To analyze the effectiveness of the second attempt ERCP 24 hours (second day) after primary failure.

Methodology: This analytical study was conducted to check the outcomes of second attempt ERCP in patients with prior failed cannulation, from June 20, 2023, to November 20, 2023, at the Department of Gastroenterology, Lady Reading Hospital, Peshawar. Patients of either sex, aged >16 years with failed biliary cannulation, and who were otherwise clinically stable were included in the study. Patients with surgically modified anatomy, an unidentified main duodenal papilla, or a history of sphincterotomy at another setup were excluded. Outcomes were assessed in terms of gaining deep biliary access (cannulation) using a therapeutic duodenoscope and endoscopy system supported by a fluoroscope while using a wire-guided sphincterotome. Factors linked to second ERCP cannulation success or failure were analyzed using SPSS version 24.

Results: Ninety-four patients were enrolled including 61 (64.9%) males and 33 (35.10%) females. The mean age of the participants was 39.01±14.831 years. The most common indication for the intervention was CBD stones, which were present in 70 (74.5%) patients. Successful cannulation on the second attempt was achieved in 72 (76.6%) patients. Experienced endoscopists achieved a greater proportion of successful cannulation (86.8%) compared to 33.3% by endoscopists with lower experience (p-value: <0.001). Logistic regression analysis was conducted to predict the outcomes (cannulation), which revealed an odds ratio for endoscopist experience of 33.604 (95% confidence interval: 6.948-162.52).

Conclusion: A second ERCP attempt 24 hours after the primary failed attempt appears to be the best course of action for the majority of clinically stable patients.

## Introduction

Endoscopic retrograde cholangiopancreatography (ERCP) is a minimally invasive intervention that has established itself as the gold standard therapeutic option for various pancreaticobiliary conditions [1]. Nonetheless, there are times when ERCP is ineffective, even when performed by highly trained professionals or at major healthcare institutions [2].

It is common knowledge that achieving selective biliary access is essential for successfully completing ERCP and determining the most effective treatment for pancreaticobiliary illness. According to the findings of previous studies, between 5% and 15% of patients who required ERCP had bile duct cannulation failure [3]. Patients with prior surgery leading to altered anatomy and those who present with duodenal papillary variations or abnormalities such as stenosis, small size, or those located within or at the border of the diverticulum are more likely to have unsuccessful biliary cannulation [4].

In case of ERCP failure on the first attempt, alternative choices include redoing the procedure (on the second attempt after 24 h) or moving on to more proficient endoscopic methods such as endoscopic ultrasound (EUS) or radiology-aided techniques (rendezvous procedures), totally percutaneous approaches, or surgical treatments [4,5]. However, the intrusive nature of these technology solutions, the dearth of interventional radiologists, and the greater morbidity and mortality rates are two of the downsides of these solutions [6]. Furthermore, there is no consensus on how to treat patients whose first ERCP ended in failed biliary cannulation, and issues with a second ERCP, such as the appropriate interval time and risk factors for cannulation failure, need to be addressed [7,8].

Despite being a safe and effective approach, biliary cannulation may not be performed on the first attempt in certain ampullary/papillary and periampullary scenarios, as well as in cases where mechanical damage caused by equipment is present [9]. When an ERCP fails, some endoscopists delay the procedure and use alternative methods, whereas others retry the procedure after a day or two, because after a short period of time, with the resolution of the edema and tissue necrosis, biliary cannulation tends to become possible [10].

Currently, there is a paucity of evidence on the results of second ERCP attempts following an unsuccessful first procedure. Therefore we decided to try ERCP once again because these other treatments are not available in our region, and we did not wish to subject the patient to an invasive surgical operation or the placement of a costly antegrade wire. In the event that the second attempt is successful, patients in our province will reap the benefits of a cost-effective treatment that is less invasive, with low morbidity and improved overall efficacy and safety. The purpose of this research was to evaluate the efficacy of a second ERCP effort that was performed 24 h after the first unsuccessful attempt.

## Materials and methods

Study design

This descriptive case series study was conducted at the Gastroenterology Department of the Lady Reading Hospital, Peshawar from June 20, 2023, to November 20, 2023, for a duration of six months.

Inclusion criteria

Patients of either sex, aged >16 with failed biliary cannulation, and who were otherwise clinically stable were included in the study.

Exclusion criteria

Patients with surgically modified anatomy, an unidentified main duodenal papilla, or a history of sphincterotomy at another setup were excluded from this study.

Operational definitions

Failed cannulation was defined as failure to pass the guidewire deeply into the pancreatic or common bile duct (CBD). Successful cannulation was defined as the transit of the guidewire into deep biliary radicals (secondary branches), as verified by fluoroscopy.

Sampling technique

Non-probability consecutive sampling.

Sample size 

The sample size was 94, taking the anticipated frequency of failed cannulation as 5% confidence level 95% and the margin of error as 4.5% [3].

Data collection procedure

Patients were enrolled in the institute’s indoor gastroenterology section. Data relating to the prior ERCP intervention were obtained from the ERCP documenting register and the database system (HMIS). Demographics (thorough medical history), physical exam, biochemical indices, imaging, ERCP indications, final diagnoses, and complications associated with the previous ERCP were documented.

A therapeutic duodenoscope and endoscopic equipment were used for all ERCPs, along with fluoroscopic support. Patients typically fasted for 6 h before the procedure. Intravenous midazolam was administered prior to the procedure for sedation and nalbuphine was used to manage the discomfort of the patients. The double-guidewire technique and needle-knife papillotomy were often employed for cannulation on the second attempt. In accordance with accepted practice, we employed a double-lumen pull-type sphincterotome that was already loaded with a hydrophilic guidewire. If the guidewire was inserted into the pancreatic duct more than twice, it was necessary to leave it there and employ the double guidewire technique. If the double guide wire technique failed, needle knife papillotomy was used for biliary cannulation. Papillotomies were performed using a needle knife, in which a longer incision was made upward in steps along the axis of the bile duct from the papillary orifice, followed by cannulation and sphincterotomy.

Data analysis

Categorical variables were assessed as frequencies (percentages), whereas continuous variables were recorded as medians with ranges. To evaluate the symmetric distribution of data, the skewness value was determined. Using the Mann-Whitney U test, statistical comparisons of data with non-normal distributions were made. The chi-square test or Pearson’s corrected chi-square test was used when dealing with categorical data. Unconstrained logistic regression analysis was performed to determine factors linked to second ERCP cannulation success or failure. A two-sided p-value ≤0.05 was considered to indicate statistical significance. SPSS (version 24) was used for all statistical analyses.

Ethical consideration

The Institutional Review Board of the Lady Reading Hospital approved the study through the approval reference number 794/LRH dated 20th June 2023 and ensured that it complied with all applicable ethical standards. Patient information anonymity was ensured, and all study procedures followed the guidelines outlined in the Declaration of Helsinki.

## Results

Ninety-four patients were registered in this study. The age of the patients ranged from 17 to 80 years, with a mean age of 39.01 years (standard deviation (SD) = 14.831 (39.01 ± 14.831 years); minimum and maximum age = 17 and 80 years, respectively). The median age was 34.0, meaning that at least 50% of patients were older than 34.0 years as shown in Table 1.

**Table 1:** Baseline characteristics (n = 94)

Variable	Minimum	Maximum	Mean ± SD	Median
Age (years)	17	80	39.01 ± 14.831	34.0
Hemoglobin (gm/dL)	9.7	13.5	11.65 ± 1.05	11.4
Platelets count 10^3^	117	301	195.84 ± 45.08	185.0
Total leucocytes 10^3^ (per mm^3^)	4.5	17.3	9.24 ± 2.53	8.25
Total bilirubin (mg/dL)	1.5	9.3	4.20 ± 1.93	4.0
Alanine transaminase (IU/L)	42	139	79.20 ± 25.82	78.0
Alkaline phosphatase (IU/L)	188	667	372.89 ± 121.59	365.0
Endoscopist experience (years)	1	5	3.69 ± 1.27	4.00

The minimum and maximum hemoglobin levels recorded for the patients were 9.7 gm/dL and 13.5 gm/dL, respectively, with a mean ± SD hemoglobin level of 11.65 ± 1.05 gm/dL. The median value for hemoglobin was 11.4, and the skewness value was 0.053, indicating that the data were symmetrically distributed, as presented in Table 1.

In terms of the blood cell counts, the platelet and total leucocyte counts were recorded. The platelet counts ranged from 117 × 103 to 301 × 103 cells/mm^3^. The mean platelet count was 195.84 × 103 (SD = 45.08). The median platelet count was 185.0, and the skewness value was 0.683, suggesting a symmetric data distribution. Similarly, the minimum and maximum white cell counts were 4.5 × 103 and 17.3 × 103, respectively, with a mean total leucocyte count of 9.24 × 103 ± 2.53 and a skewness value of 0.763, as shown in Table 1.

The hepatic biochemical profile included serum bilirubin, alanine transaminase (ALT), and alkaline phosphatase(ALP). The mean serum bilirubin was 4.20 ± 1.93 mg/dL, with minimum and maximum values of 1.5 and 9.3 mg/dL, respectively, as shown in Table 1.

The sex-wise distribution of patients revealed that 61 (64.9%) were male and 33(35.1%) were female as shown in Table 2.

**Table 2:** Frequencies and percentages with respect to sex, chief complaint, diagnosis, and type of ampulla (n = 94)

Variable	Sub-groups	Frequency	Percentage
Sex	Male	61	64.9
	Female	33	35.1
Chief complaint	Jaundice	61	64.9
	Pruritus	19	20.2
	Pain	14	14.9
Final diagnosis	CBD stone	70	74.5
	CBD stricture	11	11.7
	Ampullary mass	13	13.8
Ampulla type	Normal	60	63.8

Jaundice was the most frequently noted complaint, reported by 61 patients (64.9%), followed by pruritus in 19 patients (20.2%), whereas pain was the least common symptom present in only 14 participants (14.9%) as presented in Table 2.

CBD stone was the most commonly established final diagnosis, in 70 patients (74.5%), followed by CBD stricture and ampullary growth in 11 (11.7%) and 13 (13.8%) patients, respectively, as shown in Table 2.

Ampulla appeared normal in 60 patients (63.8%), 10 patients (10.6%) had intra-diverticular ampulla, 14 patients (14.9%) had protruding ampulla, and the remaining 10 patients (10.6%) had flat ampulla, as presented in Table 2.

Successful cannulation was achieved in 72 (76.6%) patients during the repeat ERCP attempt, as shown in Table 3.

**Table 3:** Outcomes of repeat ERCP cannulation

Variable	Frequency	Percentage
Successful	72	76.60
Unsuccessful	22	23.4

The demographics and clinical and laboratory parameters of the patients were examined in terms of cannulation using contingency tables. The results are presented in Table 4.

**Table 4:** Comparison of patient characteristics with respect to cannulation (n = 94)

Variable	Sub-groups	Cannulation		Total	X^2^ p-value
		Successful	Unsuccessful		
Sex	Male	46 (76.7%)	14 (23.3%)	60 (100.0%)	0.983
	Female	26 (76.5%)	8 (23.5%)	34 (100.0%)	
Age (years)	16-40	39 (73.6%)	14 (26.4%)	53 (100.0%)	0.433
	41-80	33 (80.5%)	8 (19.5%)	41 (100.0%)	
Chief complaint	Jaundice	47 (77.0%)	14 (23.0%)	61 (100.0%)	0.872
	Pruritus	15 (78.9%)	4 (21.1%)	19 (100.0%)	
	Pain	10 (71.4%)	4 (28.6%)	14 (100.0%)	
Diagnosis	CBD stones	55 (78.6%)	15 (21.4%)	60 (100.0%)	0.015
	CBD stricture	10 (90.9%)	1 (9.1%)	11 (100.0%)	
	CBD stricture	10 (90.9%)	1 (9.1%)	11 (100.0%)	
Ampulla	Mass ampulla	06 (46.2%)	7 (53.8%)	13 (100.0%)	0.248
	Normal	47 (78.3%)	13 (21.7%)	60 (100.0%)	
	Diverticular	8 (80.0%)	2 (20.0%)	10 (100.0%)	
	Flat	9 (90.0%)	1 (10.0%)	10 (100.0%)	
Platelet count	<150,000	10 (76.9%)	3 (23.1%)	13 (100.0%)	0.578
	>150,000	62 (76.5%)	19 (23.5%)	81 (100.0%)	
Hemoglobin (gm/dL)	<11	46 (78.0%)	13 (22.0%)	59 (100.0%)	0.684
	>11	26 (74.3%)	9 (25.7%)	35 (100.0%)	
Bilirubin (mg/d L)	<2	50 (79.4%)	13 (20.6%)	63 (100.0%)	0.366
	>2	22 (71.0%)	9 (29.0%)	31 (100.0%)	
Alanine transaminase (IU/L)	<50	11 (73.3%)	4 (26.7%)	15 (100.0%)	0.745
	>50	61 (72.2%)	18 (27.8%)	79 (100.0%)	
Alkaline phosphatase (IU/L)	<200	6 (86.7%)	1 (13.3%)	7 (100.0%)	0.554
	>200	66 (75.9%)	21 (24.1%)	87 (100.0%)	
Endoscopist experience	<2 years	6 (33.3%)	12 (66.7%)	18 (100.0%)	0.000
	>2 years	66 (86.8%)	10 (13.2%)	76 (100.0%)	

A binary logistic regression model was constructed to analyze the effect of various predictors on the dependent variable, that is, cannulation outcomes. In this case, successful cannulation was coded as 1 and unsuccessful cannulation as 0. The Omnibus test of the model coefficient was used to test the model fit. The model was significant, with a p-value = 0.001 (i.e., <0.05); hence, the model was a good fit. The Hosmer and Lemeshow statistics indicated a good fit, with a p-value = 0.202 (i.e., >0.05), demonstrating that the model adequately fits the data. Hence, there was no significant difference between the observed and predicted models. The values were almost equal and are elaborated in the contingency table for the Hosmer and Lemeshow tests. In the model summary, the Negelkerke R-square value was 0.425. The specificity and sensitivity of the model for predicting the outcomes (cannulation) were 54.5% and 91.7%, respectively, and the overall accuracy was 83.0%. The model exhibits good sensitivity (91.7%) among the patients who experience successful cannulation, as shown in Table 5.

**Table 5:** Classification table (n = 94)

Observed		Predicted		
		Cannulation outcomes		Percentage correct
		Unsuccessful	Successful	
Cannulation outcomes	Unsuccessful	12	10	54.5
	Successful	6	66	91.7
Overall percentage		-	-	83.0

The relationship between the predictors and outcomes (cannulation outcomes) is shown in Table 6. The odds ratio for hemoglobin, ALT, bilirubin, and endoscopist experience was >1. The odds of successful cannulation were 1.712 times greater in patients with normal hemoglobin levels than in those with low hemoglobin levels, with a 95% confidence interval (CI) of 0.444-6.611. Similarly, the odds ratio for serum bilirubin was 1.053, with a 95% CI of 0.132-8.425, indicating higher cannulation success with an increase in bilirubin. Most notably, the odds ratio for endoscopist experience was 33.604 (95% CI = 6.948-162.52), demonstrating that the success of cannulation increased with increasing endoscopist experience.

**Table 6:** Logistic regression analysis for predicting outcomes (cannulation)

Variable	Beta	Standard error	Wald test	Degree of freedom	Significance	Exp (B)	95% CI Exp (B)	
							Lower	Upper
Sex	-0.065	0.652	0.010	1	0.921	0.937	0.261	3.366
Complaints	-0.133	0.530	0.063	1	0.802	0.876	0.310	2.473
Diagnosis	-1.031	0.523	3.888	1	0.049	0.357	0.128	0.994
Ampulla	-0.163	0.339	0.230	1	0.632	0.850	0.437	1.653
Age	-0.355	0.719	0.243	1	0.622	0.701	0.171	2.869
Hemoglobin	0.538	0.689	0.609	1	0.435	1.712	0.444	6.611
Platelets count	-0.229	0.932	0.060	1	0.806	0.795	0.128	4.944
Alanine transaminase	0.563	0.829	0.462	1	0.497	1.756	0.346	8.911
Alkaline phosphatase	-1.399	1.344	1.083	1	0.298	0.247	0.018	3.439

## Discussion

The results described above provide a comprehensive description of the demographics and baseline characteristics of the study participants. This discussion will focus on significant conclusions and potential therapeutic applications as we delve deeper into the implications and importance of the data. The majority of pancreaticobiliary disorders are now diagnosed and treated with ERCP, which is a minimally invasive procedure. Studies have shown that experienced endoscopists using a needle-knife have improved the cannulation success rate to between 85% and 99%; however, the matter of selecting the best substitute among the available options in the event of initial ERCP failure still holds interest [1,11]. A subsequent ERCP within a few days is an effective and safe therapy for patients who are clinically stable, with a success rate of 68%-79% [2,12,13]. The results of the current study reveal an overall success rate of 76.6%, which supports the validity and vitality of a second ERCP.

Papillary edema and swelling are frequently caused by numerous ERCP attempts, which makes biliary cannulation more challenging. The probability of success can be increased by performing a second ERCP a few days after the first failed procedure because papilla edema can be reduced over the course of time; however, the appropriate spacing interval remains debated [5]. While some studies have shown an extended period of four to seven days, many studies state that the span between the first unsuccessful ERCP and the second should be within the first 24-72 h [13,14]. One study found that a four-day gap period was the sole substantial factor connected to a second ERCP failure [12]. In the current study, the second ERCP was performed after a rest period of 24 h. Additionally, there was no other group in comparison with different rest periods; hence the impact of the interval between the two procedures on the outcomes is inconclusive. Although prior research has shown that the papillary edema brought on by earlier cannulation efforts and cautery invariably disappears in three to five days, the papilla may be observed after this time with a distinct look [15]. However, during the real-world experience, it was discovered that the somewhat swollen papilla in the first half of the day was sufficiently clear to allow cannulation. Thus, a prolonged gap between subsequent ERCPs remains a matter of debate.

Our knowledge of the factors leading to failed cannulation is scarce. Guideline recommendations for papillary cannulation state that endoscopist characteristics (experience) and patient factors (anatomy) both determine the chance of effective cannulation [16]. In the present study, the experience of the endoscopist varied and represented the single most dominant predictor influencing the outcomes of cannulation, with an odds ratio of 33.604 (95% CI: 6.948-162.524). Operating challenges are typically directly increased by patient factors, such as abnormal duodenal papilla and surgically altered anatomy, which may lead to a lower success rate [17,18]. However, we failed to demonstrate the effect of the appearance of ampulla on the outcomes (odds ratio: 0.850, 95% CI: 0.437-1.653)

Using logistic regression, we discovered that a baseline normal blood bilirubin concentration was associated with failure of the second ERCP (OR: 1.053, 95% CI: 0.132-8.425). Our findings suggest that endoscopists should carefully consider patients with normal serum bilirubin levels when planning a second ERCP.

Limitations

Since the sample size was small and the study was conducted at a single center, it may not be the true representative of the community, and large studies are needed to further explore the success rate of the second attempt ERCP in our local setup.

## Conclusions

A second ERCP following the failure of the first biliary cannulation appears to be quite effective. A second ERCP performed after a rest period of 24 h may be the best course of action for most clinically stable patients who had a failed initial ERCP. This approach precludes the need for more invasive procedures like surgical intervention or interventional radiology-guided procedure, reduces the length of hospital stay, and ultimately cost.
